# The influence of different storage media on Vickers hardness and surface roughness of CAD/CAM resin composites

**DOI:** 10.1007/s10856-023-06713-7

**Published:** 2023-03-18

**Authors:** Florian Fuchs, Julius Schmidtke, Sebastian Hahnel, Andreas Koenig

**Affiliations:** 1 Department of Prosthetic Dentistry and Dental Material Science, Leipzig University, Liebigstraße 12, 04103 Leipzig, Germany; 2 Department of Prosthetic Dentistry, UKR University Hospital Regensburg, Franz-Josef-Strauß-Allee 11, 93053 Regensburg, Germany

## Abstract

**Introduction:**

This study examined Vickers hardness as well as surface characteristics of different computer-aided design/computer-aided manufacturing (CAD/CAM) resin composites prior to and after storage in various media.

**Materials and methods:**

CAD/CAM resin composite blocks (Grandio Blocs (GB), Lava Ultimate (LU), Brilliant Crios (BC), Cerasmart (GC), Shofu Block HC (SB), Tetric CAD (TC), Luxacam Composite (LC); incl. different translucency variants) were prepared, polished and surface free energy was determined. The specimens were divided into four groups: dry conditions for 24 h (25 °C), demineralized water (37 °C), Pepsi Cola (37 °C) and 75% ethanol (37 °C). After seven and 28 days of storage, Vickers hardness was determined. Surface roughness was measured after the entire storage period.

**Results and discussion:**

Vickers hardness was in the range of about 150 HV for GB, around 115 HV for LU, and 80–100 HV for BC, GC, SB, TC and LC. Only minor differences *(total:* 50.2 (6.4)–56.2 (3.2) mN/m) in surface free energy could be detected. No relationship was observed between surface free energy and filler content. However, a correlation between filler content and Vickers hardness was evident. Artificial aging caused a decrease of Vickers hardness (up to −40 HV or 35%) depending on storage media, duration and material. The changes in surface texture after immersion in different media were below a value of ΔSa = 0.015 µm.

**Conclusion:**

Artificial aging of CAD/CAM resin composites leads to a significant decrease of Vickers hardness for most materials, while only small changes in surface roughness were identified.

**Graphical Abstract:**

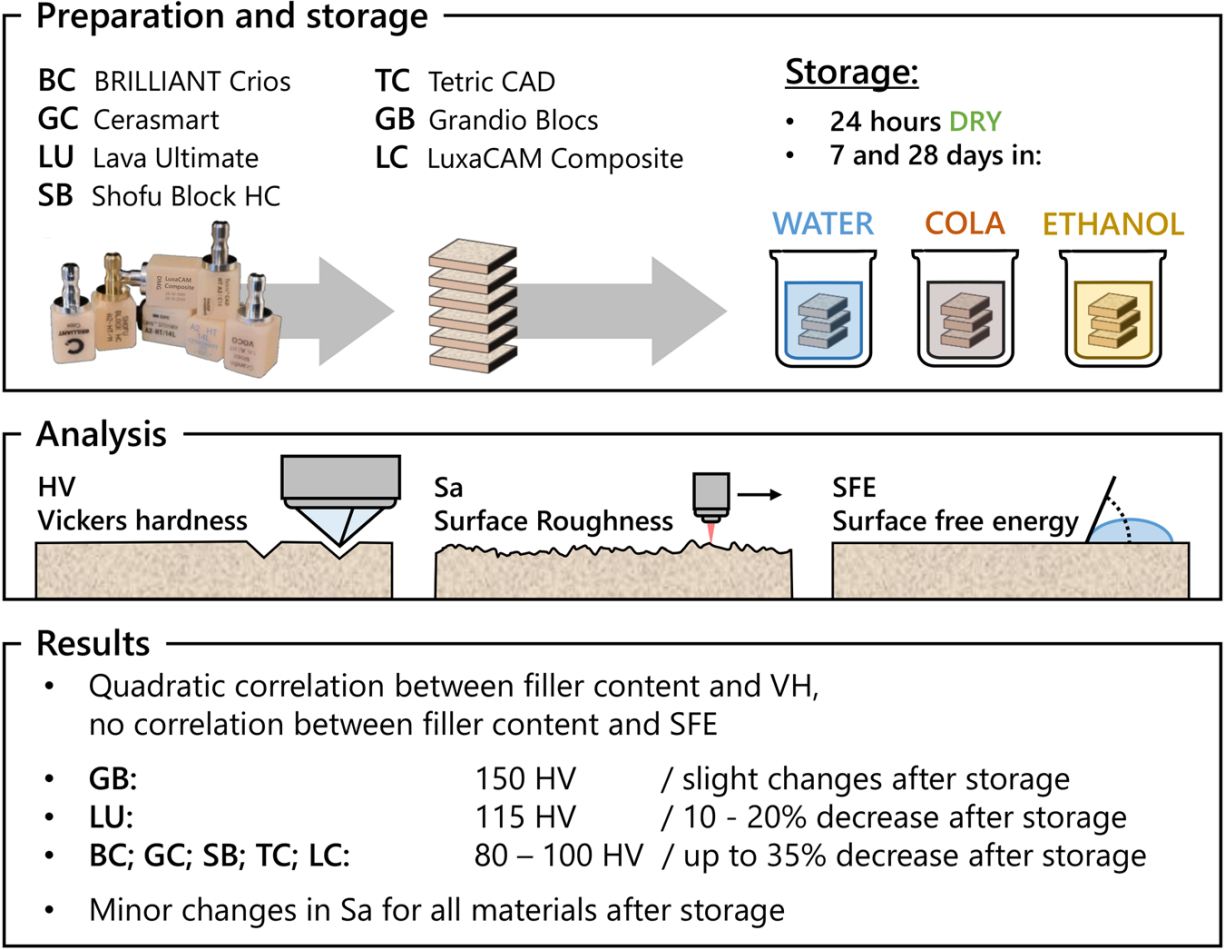

## Introduction

For the fabrication of tooth-colored, monolithic restorations, indirect resin-based composites (RBC) are an alternative to commonly used ceramics [1, 2]. In contrast to chair-side polymerizing direct resin-based composites, CAD/CAM (computer-aided design/computer-aided manufacturing) resin composites (synonym for indirect RBC) are industrially manufactured and milled after the digital design of the restoration [1]. Polymerization under high temperature and high pressures under industrial conditions produces higher polymerization rates than in direct composites (over 90%) [2]. This results in improved mechanical properties (flexural strength, hardness, density) [3] as well as reduced biofilm formation on the surface of the materials [4].

CAD/CAM resin composites consist of an organic resin matrix, inorganic fillers, and a bonding agent [1, 5]. The inorganic filler particles used in this process have a mostly amorphous character with a mass fraction of 62–83% [6]. Based on Ferracane’s classification, they are classified as “midfill hybrid composites” [7]. Additional sintering after the milling process as necessary for the most of ceramic materials is not required for CAD/CAM resin composites [2], which relevantly reduces laboratory time.

With regard to Vickers hardness (VH), Young’s modulus (E), and brittleness, in contrast to commonly used silicate ceramics (VH: 452.9–595.1; E = 61.0 GPa–67.2 GPa) CAD/CAM resin composites (VH: 62.2–102.3; E = 12.1 GPa–16.0 GPa) exhibit mechanical properties comparable to those of those of natural teeth (enamel: VH = 313.3 (22.7); E: 59.7 (13.0) GPa; dentin: VH: 62.3 (3.3); E: 16.5 (2.3) GPa) [8–10]. Especially in patients with parafunctions, these properties might help to reduce abrasion of the restoration as well as antagonists [11–13]. The fracture strength of crowns fabricated from different CAD/CAM resin composites has been reported to be similar to crowns fabricated from lithium disilicate ceramics and sufficient for molar bite force [14].

Since the application of CAD/CAM resin composites as dental materials for the fabrication of monolithic dental restorations is a rather novel approach, there is a lack of studies that investigate the clinical long-term behavior of corresponding restorations [15]. Previous results show a survival rate of 100% after one year for posterior onlays [16] and a clinical success rate of 95.0% after twelve months and 85.7% after 24 months for partial crowns [17]. A retrospective study over three years with CAD/CAM resin composite crowns reported a survival rate up to 96.4% [18]. As a result of nutrition, dental materials are exposed to different environments and must tolerate temperature fluctuations. The influence of such factors on the survival rate of restorations has been proven by various studies [19–21]. Especially for direct composites, a relevant influence of various media on the mechanical properties of the materials has been identified. For instance, both a decrease in Vickers hardness and an increase in surface roughness due to artificial aging by immersion in water or water-ethanol mixtures have been reported [22–24]. In particular, the chemical influence of ethanol by a degradation of the resin matrix and a disintegration of filler-silan-bonds have also been described [25–27].

These aging effects are reduced in materials with high filler/matrix proportions as well as those with a hydrophobic resin matrix [9, 22]. Although degradation of glass fillers due to storage in solvents has been proven [28], this effect seems to be subordinate [29]. By comparing several in vitro and in vivo studies, it was shown that storage in ethanol is associated with both a decrease in flexural strength and clinical performance in the form of chipping and/or fracture, or loss of anatomical form due to wear [27]. However, such correlations could not be identified for storage in water—although it is widely used as medium for simulating aging processes [22, 23].

Furthermore, a decrease in the fracture load of crowns made of CAD/CAM resin composites due to thermocycling [30] and a decrease in the flexural strength of CAD/CAM resin composites after storage in water (37 °C) for 30 days have been reported [31]. Acidic beverages such as Coca Cola also caused a reduction of Vickers hardness after 7 and 28 days of exposure, and CAD/CAM resin composites were more affected than ceramic materials. However, due to an underrepresentation of commercially available CAD/CAM resin composites, no correlation with physical properties could be drawn in this regard [32]. Furthermore, Ilie (2019) showed that accelerated aging has a higher impact on micromechanical properties such as Vickers hardness or indentation modulus than macroscale effects in terms of clinical performance such as chipping [33].

Microorganisms organized in oral biofilms are closely associated with the development of caries [34], gingivitis, periodontitis [35], and spreading infections [36]. Therefore, it is of clinical importance to minimize the formation of biofilm on the surface of dental restorations. High surface roughness promotes microbial adhesion [37, 38] by increasing the contact area between the organisms and the restoration surface [39] and reducing shear forces that result from salivary flow [40]. In the past, an Ra value (arithmetic mean height) of less than 0.2 µm has been established as a widely accepted threshold below which no significant further reduction in biofilm accumulation can be achieved by additional polishing [40, 41]. In this context, high surface roughness favors initial attachment of cells [42], while further growth and colonization of the biofilm occurs regardlessly of surface texture. With regard to RBCs, a relationship between roughness and biofilm formation is evident [43, 44]. Indirect composites generally showed less microbial attachment compared to direct composites under shear forces, but not under closed culture conditions [4].

For CAD/CAM resin composites, there are only few studies investigating the effects of artificial aging on mechanical properties, and the impact of different RBC formulations in materials of different manufacturers or the influence of translucency variants have largely been neglected [9, 33, 45]. Against this background, the present study investigates the surface characteristics of commercially available CAD/CAM resin composites by surveying vickers hardness, surface texture, surface free energy as well as potential correlations and their dependency on exposure to different aging media. The null hypotheses (*H*_*0*_) were as follows:

H_0_ (a) There is no difference between CAD/CAM resin composites with different translucencies in terms of Vickers hardness and surface energy.

H_0_ (b) There is no correlation between Vickers hardness or surface free energy and a previously investigated filler content of CAD/CAM resin composites [6].

H_0_ (c) There is no loss in Vickers hardness after aging in different storage media for the different CAD/CAM resin composites.

H_0_ (d) There is no difference in surface roughness after aging in different storage media for the different CAD/CAM resin composites.

## Materials and methods

### Materials

Different commercially available CAD/CAM resin composite blocks were used (Table 1). In order to compare the dependency of the individual performance of the materials on the grade of translucency, low/medium- (LT/MT) as well as high-translucency (HT) variants were analyzed where available.

**Table 1:** Product information of the materials used; the information provided by the manufacturers has been reported unless indicated otherwise

Material	Code	Manuf.	Transl.-variant	LOT	Composition		Filler content
					Organic	Inorganic	
BRILLIANT Crios	BC	COLTENE	A2 HT A2 LT	I44747 IO3O77	cross-linked methacrylates	barium glass (<1.0 µm) amorphous silicia (<20 nm)	69.4–69.8 wt.% [6]
Cerasmart^TM^	GC	GC Europe	A2 HT A2 LT	1809051 1710041	Bis-MEPP, UDMA, DMA [33]	Si, Al, Ba amorphous phases [6]	64.5–64.8 wt.% [6]
Lava^TM^ Ultimate	LU	3M^TM^ ESPE^TM^	A2 HT A2 LT	N987419 N401476	Bis-GMA, UDMA, Bis-EMA, TEGDMA [33]	silicia nanomers (20 nm) zirconia nanomers (4–11 nm) zirconia-silicia-nanoclusters (0.6–10 µm)	72.0–72.3 wt.% [6]
SHOFU Block HC	SB	Shofu	A2 HT A2 LT	071601 0818225	UDMA, TEGDMA [33]	Si, Zr, amorphous phases [6]	61.6–62.5 wt.% [6]
Tetric^®^ CAD	TC	Ivoclar Vivadent	A2 HT A2 MT	W90501 Y50470	cross-linked dimethacrylate (Bis-GMA, Bis-EMA, TEGDMA, UDMA)	barium aluminum silicate glass (<1 µm) silicon dioxide (<20 nm)	69.3–69.9 wt.% [6]
Grandio^®^ blocs	GB	VOCO	A2 HT A2 LT	1831230 1842286	methacrylates [33]	Si, Al, Ba amorphous phases [6]	82.3–83.1 wt.% [6]
LuxaCAM Composite	LC	DMG	A2	795497	highly networked polymer	silicate glass filler	68.8 wt.% [6]

### Methods

#### Sample preparation

The CAD/CAM blocks were cut into rectangular slices (*n* = 104) with a thickness of 3 mm using the IsoMet®4000 Linear Precision saw (Buehler Ltd., Lake Bluff, IL, USA) under constant water irrigation (according to ISO 6872) [46]. All specimens were polished using a semi-automatic polishing unit “Pedemin-2/DAV-5” (Struers GmbH, Willich, Germany) and a standardized polishing regime employing silicon carbide papers with successively decreasing grain size (P220, P500, P1200, P2000, P4000) under water cooling and 300 rpm for 15 s, respectively. The samples were then cleaned in an ultrasonic bath (Bandelin electronic GmbH & Co. KG, Berlin, Germany) with demineralized water for ten minutes and randomized into four groups:

Group 1: Specimens were stored under dry conditions for 24 h at room temperature. Subsequently, Vickers hardness and surface roughness was determined.

Group 2: Specimens were stored in demineralized water at 37 °C.

Group 3: Specimens were stored in 75% ethanol/ demineralized water solution at 37 °C.

Group 4: Specimen were stored in Pepsi Cola at 37 °C.

In groups 2–4, Vickers hardness was measured after seven days and 28 days of storage and surface roughness after the entire storage period of 28 days.

#### Vickers hardness

Vickers hardness (VH) was determined with a microhardness tester (MHT-4, Anton Paar Group AG, Graz, Austria) in combination with research light microscope (Microphot-FXA, Nikon Corp., Tokio, Japan) under a 0.2 kg loading and 12 s dwell time (HV 0.2 according to DIN EN ISO 6507-1). For each material and time, fifteen indentations were analyzed (*n* = 15), where the distance between the center of two indentations was at least five times the indentation diagonal length. Diagonals were measured using a confocal laser scanning microscope (see “surface roughness”) and averaged. Vickers hardness was calculated according to DIN EN ISO 6507-1 using the formula [47]:

1 $$HV \approx 0.1891 \cdot {\rm{F}} \cdot {\rm{d}}^{ - 2}$$

F = testing force in N; d = average diagonal length of indentation in mm.

#### Surface roughness

Confocal laser scanning microscopy (VK-X1000/X1050, KEYENCE, Osaka, Japan) with a ×50 magnification (CF IC EPI Plan 50x; *NA* = 0.8; *WD* = 0.54 mm), a red laser (λ = 661 nm), and a resolution of 1024 × 768 pixels was used for surface analysis. Measurements of surface and indentation diagonals were performed by “VK Viewer 1.1.2.174” software (Keyence Cooperation, Osaka, Japan). The surface data was subjected to a S-Filter of 0.8 µm and a L-Filter of 0.1 mm (filter-type: double Gaussian) and analyzed for arithmetical mean height (Sa) according to DIN EN ISO 25178-2 using the software “MultiFileAnalyzer” 2.1.3.89 (Keyence Cooperation, Osaka, Japan) [48].

#### Surface free energy

The surface free energy of all materials was measured using highly polished surfaces (Sa <0.02 µm). Contact angle measurements were performed by a DSA25S (Krüss, Hamburg, Germany) using purified water and diiodomethane. Eight measurements were performed for each test liquid, each with a drop volume of 0.2 µl and a time interval of 30 s between application and measurement at 23 °C under air atmosphere [49]. The application was software-controlled using the manufacturer’s own DO3252 “Liquid Needle” dosing unit. Analyses were performed with the software “ADVANCE 1.11” (Krüss, Hamburg, Germany) by averaging the contact angles on both sides (fitting method: ellipse) and surface free energy (total, dispersive and polar parts) was calculated according to Owens and Wendt [50].

#### Statistics

All data were analyzed for normal distribution according to Shapiro-Wilk. Differences in Vickers hardness and surface free energy between the materials and their manufacturer-specific translucency variants as well as the differences in Sa parameters were firstly checked for homogeneity of variances using the Levene test. In case of equality of variances, a one-way analysis of variance (ANOVA) and Bonferroni post-hoc tests were performed. Assuming inhomogeneous variances, statistical analysis was performed by Welch ANOVA followed by Dunnett-T3 post-hoc multiple comparisons. A possible correlation between Vickers hardness or surface free energy and the filler content of the CAD/CAM resin composites was investigated using a test for Pearson correlation and a quadratic regression with previously published data from our research group regarding the study of filler content of CAD/CAM resin composites [6]. Differences in mean values between the initial values for Vickers hardness depending on the storage medium and storage time were examined using a *t* test. A two-way ANOVA with Bonferroni post-hoc tests were used for statistical analysis of the influence of medium and time factors. Significance level was set to α = 0.05.

## Results

**Fig. 1:**
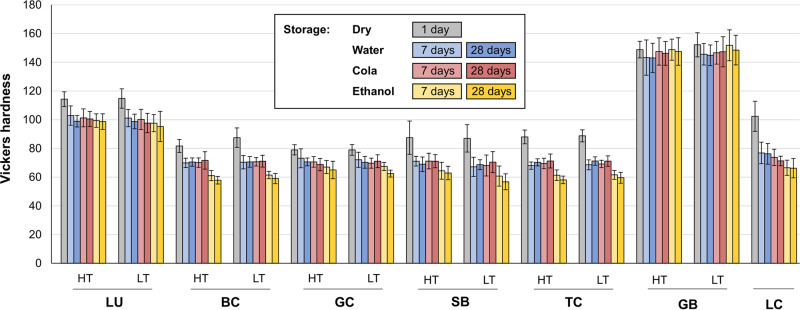
Vickers hardness of the tested samples (means and standard deviations are indicated) and their manufacturer-specific translucency variants both after dry storage and after storage in the various media for seven and 28 days

After 24 h of dry storage, GB showed the highest (*p* < 0.001) Vickers hardness values with 148.8 (2.9)–152.2 (4.2) HV, followed by LU with 114.3 (2.6)–114.8 (3.4) HV, and LC with 102.4 (5.2) HV. The Vickers hardness of all other specimens (BC, GC, SB, TC) ranged between 79.0 (1.9) HV and 88.8 (2.1) HV (Table 2). No significant differences were identified between BC-LT and TC, BC-HT and GC, BC and SB as well as SB and TC (Fig. 1). With the exception of BC, no significant differences in Vickers hardness could be detected between the respective translucency variants within the samples of a single manufacturer (see Supplementary information, Table S3).

**Table 2:** Mean values and standard deviations (SD) of Vickers hardness (HV) for all tested materials

Spec.		Reference	Demineralized water				Cola				Ethanol			
			7d	*p* value	28d	*p* value	7d	*p* value	28d	*p* value	7d	*p* value	28d	*p* value
**LU**	HT	114.3 (2.6)	102.9 (3.4)	<0.001	99.0 (2.0)	<0.001	101.3 (3.1)	<0.001	100.5 (2.6)	<0.001	99.4 (2.4)	<0.001	98.7 (2.7)	<0.001
	LT	114.8 (3.4)	101.1 (3.0)	<0.001	98.8 (2.6)	<0.001	100.2 (3.5)	<0.001	97.7 (3.4)	<0.001	97.6 (3.0)	<0.001	95.3 (5.2)	<0.001
**BC**	HT	81.7 (2.3)	69.9 (1.6)	<0.001	70.5 (1.4)	<0.001	70.1 (1.6)	<0.001	71.7 (3.0)	<0.001	61.1 (1.8)	<0.001	57.8 (1.3)	<0.001
	LT	87.5 (3.4)	70.4 (2.3)	<0.001	70.7 (1.9)	<0.001	70.7 (1.5)	<0.001	71.1 (2.1)	<0.001	61.5 (1.3)	<0.001	59.0 (1.8)	<0.001
**GC**	HT	79.0 (1.8)	73.2 (3.3)	<0.001	70.6 (1.4)	<0.001	70.6 (1.8)	<0.001	68.7 (2.1)	<0.001	67.0 (2.3)	<0.001	65 (3.1)	<0.001
	LT	79.0 (1.9)	72.1 (2.6)	<0.001	70.3 (2.0)	<0.001	69.7 (1.7)	<0.001	71.1 (2.2)	<0.001	67.5 (1.4)	<0.001	62.5 (1.2)	<0.001
**SB**	HT	87.5 (5.8)	71.1 (1.7)	<0.001	69.0 (2.5)	<0.001	71.2 (2.7)	<0.001	71.1 (2.3)	<0.001	64.4 (2.9)	<0.001	62.9 (2.4)	<0.001
	LT	87.0 (4.8)	67.2 (3.3)	<0.001	68.7 (1.7)	<0.001	68.1 (3.5)	<0.001	70.4 (3.7)	<0.001	60.7 (3.5)	<0.001	56.8 (2.8)	<0.001
**TC**	HT	88.0 (2.4)	68.0 (1.3)	<0.001	70.3 (1.2)	<0.001	69.5 (1.8)	<0.001	71.2 (2.4)	<0.001	61.3 (1.8)	<0.001	58.1 (1.3)	<0.001
	MT	88.8 (2.1)	68.6 (1.7)	<0.001	71.2 (1.4)	<0.001	69.2 (1.3)	<0.001	71.2 (1.8)	<0.001	61.6 (1.5)	<0.001	59.6 (1.9)	<0.001
**GB**	HT	148.8 (2.9)	143.3 (6.1)	0.006	143.1 (5.1)	0.002	147.5 (4.8)	0.428	146.2 (4.1)	0.031	148.8 (3.7)	0.997	147.5 (4.8)	0.398
	LT	152.2 (4.2)	145.6 (3.7)	0.001	144.9 (3.6)	<0.001	146.6 (4.0)	0.004	147.3 (5.2)	0.028	151.8 (5.4)	0.821	148.5 (5.1)	0.042
**LC**	–	102.4 (5.2)	76.9 (3.7)	<0.001	76.2 (3.6)	<0.001	73.8 (2.8)	<0.001	71.3 (1.7)	<0.001	66.6 (2.6)	<0.001	66.2 (3.3)	<0.001

The *p* values for statistical analysis between the reference HV and the HV after storage in different media showed no difference for different durations

Storage in demineralized water, cola, and ethanol resulted in a strongly significant reduction (*p* < 0.001) of Vickers hardness for all samples except GB (Table 2). In contrast to all other specimens, the greatest decrease in Vickers hardness for GB was produced by storage in water. After exposure to ethanol, no changes in Vickers hardness for GB-HT (*p* = 0.398) and minor changes for GB-LT (*p* = 0.042) were measured.

Two-way ANOVA indicated that for BC, GC, SB, TC, and LC ethanol immersion caused the most prominent decline in Vickers hardness, which was significantly higher than for both demineralized water and cola (*p* < 0.001, respectively). For LU and GC, the duration of storage was also a significant factor for the observed decline in Vickers hardness (see supplementary information, Table S2).

**Fig. 2:**
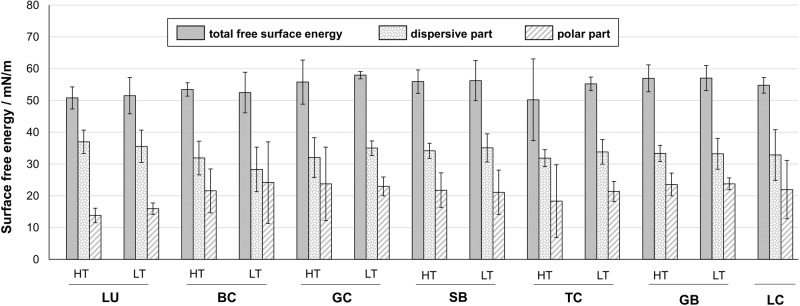
Surface free energy (with standard deviations) and its polar/dispersive parts (according to the Owens/Wendt approach [50])

Surface free energy of the specimens ranged between 50.2 (6.4) and 58.0 (0.6) mN/m. While the dispersive parts were between 28.3 (3.5) and 37.0 (1.8) mN/m, the polar parts were spread wider and ranged between 13.8 (1.1) and 24.2 (6.4) mN/m (Fig. 2). No differences could be identified between the translucency variants within materials from the same manufacturer (supplementary information, Table S1). The lowest polar fractions were identified for LU.

A test for Pearson correlations showed a significant correlation between the filler content of the CAD/CAM resin composites and the observed Vickers hardness, but not for the surface free energy (Table 3). An additional quadratic regression resulted in a coefficient of determination of *R²* = *0.889*. Based on the surface free energy results, it is qualitatively hypothesized, taking into account the study design, that no other correlation is present (see supplementary information, Fig. S1).

**Table 3:** Pearson correlation of the investigated variable Vickers hardness and surface free energy to the filler contents of the investigated CAD/CAM resin composite determined by Koenig et al. [6]

Variable in correlation to filler content	Pearson correlation coefficent r	coefficient of determination R²	*p* value
Vickers hardness	0.908	0.824	<0.001
Surface free energy			
Total	0.015	<0.001	0.960
Dispersive part	−0.029	0.001	0.926
Polar part	0.031	0.001	0.921

**Fig. 3:**
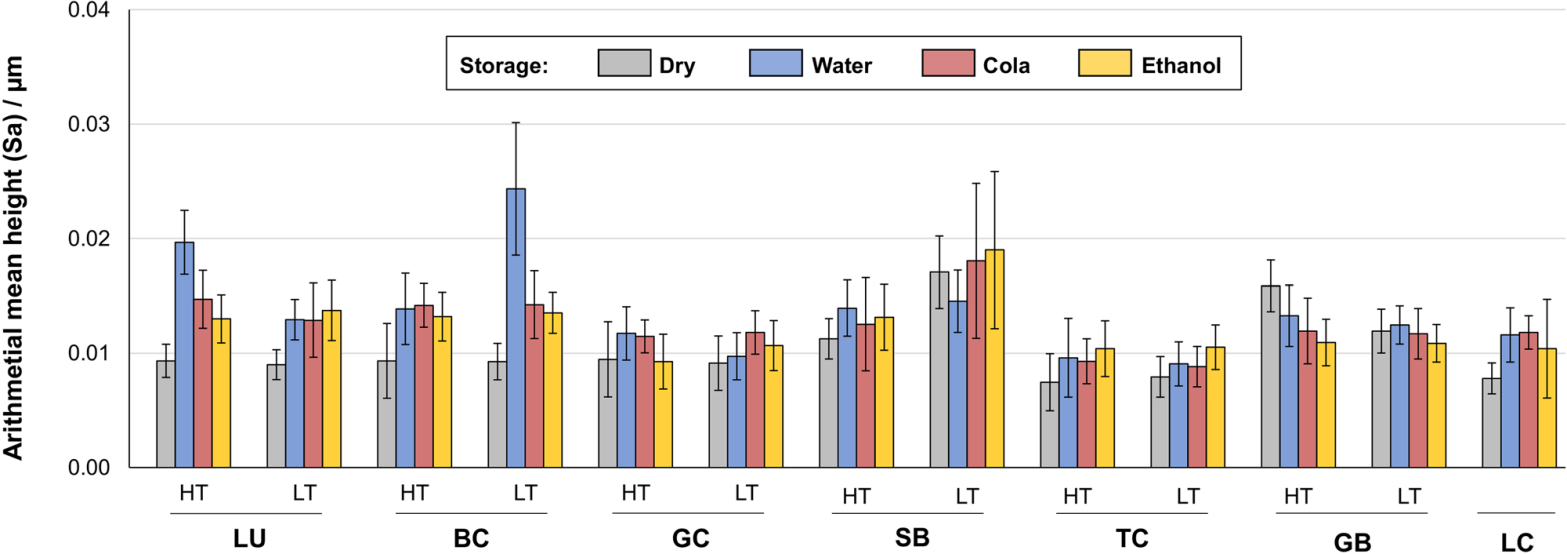
Sa (arithmetical mean height) as a description for surface roughness (mean and standard deviation) and as a function for the respective storage: dry for 24 h; water, cola and ethanol for 28 days

Regarding surface texture analysis, storage in the aging media caused a significant change in arithmetical mean height in almost all materials (Fig. 3). Highest values were observed after water storage for BC-LT and LU-HT with a change of ΔSa = 0.015 (0.003) µm, and ΔSa = 0.010 (0.002) µm, respectively. All other changes were smaller than ΔSa = 0.005 µm.

## Discussion

The results of the current study are discussed in dependence on the four (a–d) null hypotheses.

H_0_ (a) There is no difference between the CAD/CAM resin composites with different translucencies in terms of Vickers hardness and surface energy.

This null hypothesis must be partially rejected. Initially, the various CAD/CAM resin composites showed partial differences in terms of Vickers hardness and three different ranges could be distinguished: (1) GB with 150 (5) HV, (2) LU with 115 (4) HV and (3) BC, GC, SB, TC as well as LC within a range of 79 (2) – 102 (6) HV. However, with the exception of BC, Vickers hardness did not differ between the translucency variants HT/LT (or MT) in materials from the same manufacturer. For BC, differences in HT and LT should be further investigated with an expanded sample number to assess whether a type I error can be excluded.

H_0_ (b) There is no correlation between Vickers hardness or surface free energy and filler content of CAD/CAM resin composites.

The null hypothesis was partially rejected, since a correlation with the filler content of the CAD/CAM resin composites could be demonstrated for Vickers hardness, but not for surface free energy. As already described by Alshabib et al. (2019), due to the high filler content of current CAD/CAM resin composites, pronounced changes in the mechanical properties can be expected when the filler content is varied [22].

H_0_ (c) There is no loss in Vickers hardness after aging in different storage media for the different CAD/CAM resin composites.

This null hypothesis could be rejected for all materials except GB. The changes in Vickers hardness of the CAD/CAM resin composites examined in this study were 10–20% reduction for LU and up to 35% reduction for BC, GC, SB, TC, and LC except for GB. This contrasts with the small reduction in Vickers hardness of up to 3% observed in Colombo et al. (2019) for zirconia-reinforced glass ceramic when placed in cola for 28 days. According to the *p* values, it can be assumed that exposure to demineralized water has a greater influence on the Vickers hardness of GB than cola or ethanol. To exclude a type I error, further studies with a larger number of samples are recommended here.

Changes in the mechanical properties of direct composites as induced by aging have been explained by chemical and physical degradation due to diffusion-related solvent sorption. These processes cause a softening of the resin matrix and reduction of polymeric chain interactions, which produces tensile stress that affects the matrix/filler interface [51] and leads to the formation of a porous subsurface layer [52]. Furthermore, hydrolysis affects the polymer network-structure by producing oligomers and monomers [23, 24]. In addition, it results in hydrolysis of the Si-O-Si bonds between filler particles and silanes, which finally weakens the filler-matrix-interface. Ferracane and Berge [29] reported that these aging effects are caused by the organic matrix and that degradation of the filler fraction plays a minor role [29]. Accordingly, degradation processes should be minimized in materials with high filler fractions [9], which supports little changes observed for GB in the current study. Moreover, the data of the current study underline that aging with cola has no additional effect than demineralized water. This observation might indicate that there is no other relevant effect producing additional stress that results from the exposure to acid (i.e. carbonic and phosphoric acid). Aging processes were highly relevant for ethanol as aging medium; this phenomenon could be attributed to the higher solubility of the organic matrix in ethanol and a simplified penetration by ethanol as a more organophilic molecule in contrast to water-based liquids. Consequences include potential degradation of the matrix and a weakening of the bonds between matrix and filler particles due to hydrolysis of the Si-O-Si bonds [22, 23].

H_0_ (d) There is no difference in surface roughness after aging in different storage media for the different CAD/CAM resin composites.

This null hypothesis must be rejected since for almost all CAD/CAM resin composites, changes in surface texture occurred after immersion in the various media. However, since changes in the mean value of Sa were in the range of the third decimal place, potential measurement errors must also be taken into consideration and the results have to be interpreted with caution. Nevertheless, it is evident that the observed maximum changes are in a scale of ΔSa = 0.015 µm and thus below the threshold value of an Ra or Sa value of 0.2 µm [40, 41], supporting the results of Schmohl et al. (2022) regarding the acid resistance of CAD/CAM resin composites [53]. Therefore, it is assumed that no clinical relevance can be derived regarding an impact on biofilm formation. Furthermore, it should be noted that other surface characteristics that might be the result of the fabrication and curing process may have a higher influence on biofilm formation than surface roughness and surface free energy [4, 54].

### Limitations

Only changes in Vickers hardness due to storage in different media were considered in the current study. For a comprehensive evaluation of the mechanical properties, additional analyses of e.g. flexural strength and elastic modulus are recommended. No investigations were carried out on a common dental ceramic. In order to allow comparisons, further studies should consider this material as a reference in their study design. In addition, analyses of other in vitro or clinically relevant media (ethanol in lower concentration, chlorhexidine, saliva, etc.) and an investigation of an effect of the temperature could help to get a more detailed view. A potential leaching of the filler particles should be investigated, as has already been reported for direct composites after six months of storage [55]. In addition, further correlations with previous studies on filler content, distribution, and chemical composition should be considered.

## Conclusion

*H*_*0*_*(a):* Commercially available CAD/CAM resin composites differ partially in Vickers hardness (79.0 (1.9)–152.2 (4.2) HV) and surface free energy (total: 50.2 (6.4)–56.2 (3.2) mN/m; dispersive: 28.3 (3.5)–37.0 (1.8) mN/m; polar: 13.8 (1.1)–24.2 (6.4) mN/m) regardless of their respective translucency variants, except for BC.

*H*_*0*_*(b)*: For the correlation between Vickers hardness and filler content, both a Pearson and a quadratic correlation could be identified, whereas no correlation to surface free energy could be drawn.

*H*_*0*_*(c)*: The Vickers hardness of the investigated materials were reduced by immersion in different media and can be divided into the following groups:

1. Highest Vickers hardness values of 150 (5) HV as well as no or only slight changes after immersion in various media were identified for Grandio Blocs (GB).
2. Lava Ultimate (LU) showed the second highest Vickers hardness, which was 115 (4) HV, decreasing 10–20% after storage in different media.
3. Brilliant Crios (BC), Cerasmart (GC), Shofu Block HC (SB), Tetric CAD (TC), and Luxacam Composite (LC) initially featured Vickers hardness values between 79 (2) and 102 (6) HV. Artificial aging produced a decline of up to −35% in Vickers hardness.

*H*_*0*_*(d):*Artificial aging produced only little changes in surface roughness (max. ΔSa = 0.015 (0.003) µm), a clinical relevance is to be doubted.

## Supplementary information

Supplementary Information
