# Supplementary material for: The influence of different storage media on Vickers hardness and surface roughness of CAD/CAM resin composites
Source: J Mater Sci Mater Med. 2023 Mar 18;34(3):13. doi: 10.1007/s10856-023-06713-7 (PMC10024666; doi:10.1007/s10856-023-06713-7)
Supplement: Supplementary file 1 — Supplementary Information [file 10856_2023_6713_MOESM1_ESM.pdf]

The influence of different storage media on Vickers hardness and surface roughness of CAD/CAM resin composites

Tab. S1 Mean values and standard deviations (SD) for surface free energy (total), with the dispersive (disp.) and polar parts (pol.) of all tested materials (Spec.) and Translucency variants (Tran.). The column "Sign. Diff." indicates the groups with a significant difference ($p < 0.05$)

Spec.	Tran.	Surface free energy / mN/m					
		**Total**	*Sign. diff.*	**Disp.**	*Sign. diff.*	**Pol.**	*Sign. diff.*
**LU**	**HT**	51.5 (2.8)	*GC-LT; TC-MT; SB-HT/LT; GB-HT/LT; LC*	35.6 (2.5)	*BC-LT; TC-HT; GB-HT*	15.9 (0.9)	*LU-LT; BC-LT; LC; GC-LT; SB-HT/LT; TC-MT; GB-HT/LT*
	**LT**	50.8 (1.7)	*GC-LT, GB-HT GB-LT*	37.0 (1.8)	*BC-LT*	13.8 (1.1)	*LU-HT, GC-LT SB-HT, TC-MT GB-HT/LT*
**BC**	**HT**	53.5 (1.0)	*GC-LT, GB-LT*	31.9 (2.7)	-	21.6 (3.5)	-
	**LT**	52.5 (3.2)	*GC-LT*	28.3 (3.5)	*LU-HT/LT, GC-LT SB-HT/LT*	24.2 (6.4)	*LU-HT*
**GC**	**HT**	55.8 (3.5)	-	32.1 (3.1)	-	23.8 (5.8)	-
	**LT**	58.0 (0.6)	*LU-HT/LT; BC-HT/LT TC-MT; LC*	35.0 (1.2)	*BC-LT TC-HT*	22.9 (1.5)	*LU-HT/LT*
**SB**	**HT**	55.9 (1.9)	*LU-HT*	34.2 (1.2)	*BC-LT*	21.8 (2.7)	*LU-HT/LT*
	**LT**	56.2 (3.2)	*LU-HT*	35.1 (2.2)	*BC-LT*	21.1 (3.5)	*LU-HT*
**TC**	**HT**	50.2 (6.4)	-	31.9 (1.3)	*LU-HT; GC-LT*	18.3 (5.7)	-
	**MT**	55.2 (1.1)	*LU-HT; GC-LT*	33.8 (1.9)	-	21.4 (1.6)	*LU-HT/LT*
**GB**	**HT**	57.0 (2.1)	*LU-HT/LT*	33.4 (1.3)	*LU-HT*	23.6 (1.8)	*LU-HT/LT*
	**LT**	57.0 (2.0)	*LU-HT/LT; BC-HT*	33.2 (2.4)	-	23.8 (0.9)	*LU-HT/LT*
**LC**	**-**	54.8 (1.2)	*LU-HT; GC-LT*	32.8 (4.0)	-	21.9 (4.6)	*LU-HT*

Tab. S2 P-values by two-way ANOVA of all tested materials for examining the significance of media (demineralized water; cola; ethanol) and duration (7 d; 28 d) of storage

**Specimen and translucency variant**		*two-way ANOVA*		
		**Media**	**Duration**	**Media*Duration**
**LU**	HT	*0.029*	*0.001*	*0.028*
	LT	*< 0.001*	*0.022*	*0.995*
**BC**	HT	*< 0.001*	*0.440*	*< 0.001*
	LT	*< 0.001*	*0.058*	*0.002*
**GC**	HT	*< 0.001*	*0.002*	*0.870*
	LT	*< 0.001*	*0.001*	*< 0.001*
**SB**	HT	*< 0.001*	*0.050*	*0.203*
	LT	*< 0.001*	*0.948*	*0.003*
**TC**	HT	*< 0.001*	*0.484*	*< 0.001*
	MT	*< 0.001*	*0.094*	*< 0.001*
**GB**	HT	*< 0.001*	*0.367*	*0.902*
	LT	*0.002*	*0.185*	*0.307*
**LC**		*< 0.001*	*0.050*	*0.243*

Tab. S3 P-values of the multiple comparisons of Vickers hardnesses among all samples after 24 h of dry storage, performed by Welch-ANOVA (determined by Levene test) and Dunnett-T3-post hoc test (H_0: No differences between the tested groups)

		LC	GB		TC		SB		GC		BC		LU
		-	LT	HT	MT	HT	LT	HT	LT	HT	LT	HT	LT
LU	HT	*< 0.001*	*< 0.001*	*< 0.001*	*< 0.001*	*< 0.001*	*< 0.001*	*< 0.001*	*< 0.001*	*< 0.001*	*< 0.001*	*< 0.001*	*1.000*
	LT	*< 0.001*	*< 0.001*	*< 0.001*	*< 0.001*	*< 0.001*	*< 0.001*	*< 0.001*	*< 0.001*	*< 0.001*	*< 0.001*	*< 0.001*	
BC	HT	*< 0.001*	*< 0.001*	*< 0.001*	*< 0.001*	*< 0.001*	*0.054*	*0.107*	*0.083*	*0.083*	*< 0.001*		
	LT	*< 0.001*	*< 0.001*	*< 0.001*	*1.000*	*1.000*	*1.000*	*1.000*	*< 0.001*	*< 0.001*			
GC	HT	*< 0.001*	*< 0.001*	*< 0.001*	*< 0.001*	*< 0.001*	*< 0.001*	*< 0.001*	*1.000*				
	LT	*< 0.001*	*< 0.001*	*< 0.001*	*< 0.001*	*< 0.001*	*< 0.001*	*< 0.001*					
SB	HT	*< 0.001*	*< 0.001*	*< 0.001*	*1.000*	*1.000*	*1.000*						
	LT	*< 0.001*	*< 0.001*	*< 0.001*	*1.000*	*1.000*							
TC	HT	*< 0.001*	*< 0.001*	*< 0.001*	*1.000*								
	MT	*< 0.001*	*< 0.001*	*< 0.001*									
GB	HT	*< 0.001*	*0.566*										
	LT	*< 0.001*											

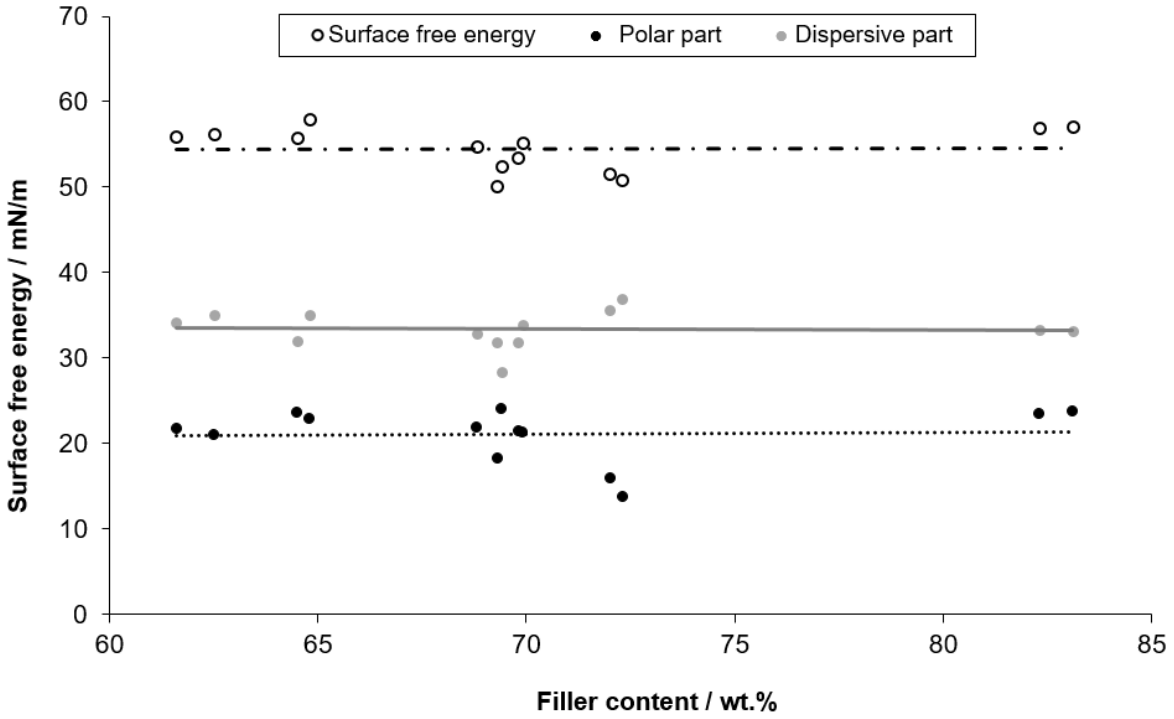

Fig. S1 Linear regression and evaltuation for correlation of the determined values of surface free energy and filler content of the investigated CAD/CAM resin composites according to Koenig et al. (2021) [6]
